# The impact of personality traits on the return of major depression: a case–control study

**DOI:** 10.3389/fpsyg.2025.1454673

**Published:** 2025-03-31

**Authors:** Nada Altaweel, Rachel Upthegrove, Steven Marwaha

**Affiliations:** ^1^Institute for Mental Health, School of Psychology, University of Birmingham, Birmingham, United Kingdom; ^2^Department of Psychology, Princess Nourah Bint Abdulrahman University, Riyadh, Saudi Arabia; ^3^Birmingham Woman's and Children's NHS Foundation Trust, Birmingham, United Kingdom; ^4^Department of Psychiatry, University of Oxford, Oxford, United Kingdom; ^5^Specialist Mood Disorders Clinic, Birmingham and Solihull Mental Health NHS Foundation Trust, Birmingham, United Kingdom

**Keywords:** personality traits, depression, emotional dysregulation, affective lability, impulsivity

## Abstract

**Background:**

Major depression is a common, chronic, recurrent, debilitating disorder. Despite effective treatments, remission rates remain low, and many of those who do experience remission then relapse. Some personality traits are potential risk factors for relapse, though they have, to date, received insufficient attention. There is growing attention to the role of emotional dysregulation in recurrent depression. We aimed to investigate the association between the return of major depression and emotional dysregulation, affective lability, and impulsivity personality traits.

**Method:**

A case–control design sampling adults over 18 years old with a history of depression and currently either experiencing a depressive episode (cases) or currently being free of a depressive episode (controls). Current depression was assessed using the Patient Health Questionnaire-9, and study participants were recruited online. Multi-staged logistic regression modelling was used to explore the association between personality traits and the return of depression, adjusting for important confounding factors.

**Results:**

One hundred fifty two respondents (76 cases and 76 controls) were recruited. Emotional dysregulation was significantly associated with the return of depression (OR = 1.03, 95% CI [1.00–1.06], *p* = 0.04) even after adjustment for the confounding factors: marital status and childhood trauma. Childhood trauma (OR = 1.04, 95% CI [1.00–1.08], *p* = 0.03) and being widowed, divorced, or separated (OR = 13.95, 95% CI [1.16–166], *p* = 0.03) were also associated with the return of depression. Our analysis did not detect any association between affective lability and impulsivity and the return of depression.

**Limitations:**

Our study relied on self-report questionnaires, including measuring depression. We used cross-sectional data in the present study analysis.

**Conclusion:**

Our findings suggest emotional dysregulation and childhood trauma could work as risk factors and predate depression. This information can be used to develop targeted treatment plans and improve therapeutic outcomes.

## 1 Introduction

Major depressive disorder (MDD) is one of the most common mental disorders worldwide (Liu et al., 2020). A considerable need remains to improve the outcomes of its treatments, as it is a highly recurrent disorder. Despite effective treatments, around 50% of individuals who have experienced a single depressive episode and recovered are prone to experiencing one or more episodes at any point during their lives, and up to 80% of individuals who have had more than two episodes will experience further episodes within 5 years (Burcusa and Iacono, 2007).

The return of depression can be described by the terms relapse and recurrence. Frank et al. (1991) proposed an operational criterion grounded in consistent, empirical evidence for each term that reflects a response to the course of depression. They characterised *relapse* as a return of depressive symptoms following partial remission but prior to full recovery. The term *recurrence* was described as “the emergence of a depressive episode after a sufficient period of remission to assume that recovery had occurred” (Frank et al., 1991). Both terms can be described as a return of depression.

Research has increasingly highlighted the role of personality traits, particularly those related to negative emotionality, in predisposing individuals to these outcomes. Negative emotionality, encompassing traits such as emotional instability, high sensitivity to stress, and a propensity for negative affect, has been linked to an elevated risk of relapse and recurrence due to its impact on cognitive and emotional regulation (Ormel et al., 2013). These traits can amplify stress reactivity and maladaptive coping, leading to sustained vulnerability even after remission (Clark et al., 2020). Moreover, personality dimensions such as neuroticism, a core component of negative emotionality, have been consistently associated with the return of depressive episodes, as they predispose individuals to rumination and pessimistic interpretations of life events (Barnhofer and Chittka, 2010). Recognising these links provides critical insight into the development of relapse prevention strategies that address enduring personality-related vulnerabilities.

Personality traits related to emotional dysregulation, such as affective lability (AL), are important factors associated with the return of depression. Emotional dysregulation and affective lability are interrelated constructs that collectively influence emotional instability. Emotional dysregulation refers to difficulties in managing and modulating emotions, leading to intense and often inappropriate responses that can hinder effective coping (Gross, 2013). Affective lability, defined as frequent and rapid shifts between emotional states, exacerbates this instability by making emotional responses highly variable and unpredictable (Harvey et al., 1989). When emotional dysregulation is present, high affective lability may lead to an amplified sense of emotional chaos, as individuals struggle to maintain consistency in their reactions and are more prone to impulsive behaviour. This relationship is particularly evident in mood and personality disorders, where the combination of emotional dysregulation and heightened affective lability contributes to significant functional impairments and distress (Gratz and Roemer, 2004).

A meta-analytical review reported that maladaptive emotional regulation strategies among people with depression were found to continue even after recovery to represent a key risk factor for relapse (Visted et al., 2018). In addition, some evidence in the literature was presented on the positive role of involving emotional regulation skills in interventions to prevent depressive relapse. For example, a randomised controlled trial aimed to combine emotional regulation and mindfulness skills to prevent relapse of depression reported that patients who received training in emotional regulation and mindfulness skills showed a significant reduction in depressive symptoms (Elices et al., 2017).

Affective lability has also been linked to mental disorders. Marwaha et al. (2018) demonstrated in their case–control study that levels of affective lability were higher in individuals with mental disorders compared to those without mental disorders. They also showed that AL predated the onset of depression (Marwaha et al., 2018), which could contribute to understanding and preventing the course of illness.

Additionally, there are indications in the literature on impulsivity as another factor that might contribute to the return of MDD. The term impulsivity is often used to describe “actions without foresight” (Dalley et al., 2011); it is the tendency to act prematurely. Results of a study with a sample of 127 inpatients suffering from depression reported a significant correlation between impulsivity and severe depression (Corruble et al., 2003). A meta-analytical review reported a strong association between remitted depression and impulsivity that persists in remission, to be a potential risk factor for relapse (Saddichha and Schuetz, 2014). Moreover, a systematic review on personality traits and depressive relapse found that neuroticism, dependent, obsessive-compulsive, and borderline personality were associated with depressive relapse. Emotional dysregulation and impulsivity represent key features of these factors that need further attention to understand their impact on the return of depression (Altaweel et al., 2023).

Despite the efforts, some personality traits have not been sufficiently investigated regarding their role in the return of depression. In a systematic review and meta-analysis on factors associated with depressive relapse, the majority of studies have focused on clinical, demographic, and other types of factors associated with the return of depressive symptoms (Buckman et al., 2018). That review showed that only a few studies had investigated the role of personality in the relapse and recurrence of depression, and mostly that was through assessing personality disorders, not personality traits that do not meet the diagnosis level of a disorder.

What is not yet clear is the impact of personality traits on the return of depression among many depressed patients who do not have personality disorders diagnosis. This indicates the need to understand the various perceptions about what and how some personality traits can lead to a return of depression. An increased investigation of the relationship between personality pathology and the return of depression could overcome the methodological limitations in the available studies and provide further validation of the current evidence on this relationship.

Highlighting less addressed factors of the return of depression, like emotional dysregulation, affective lability and impulsivity, could help reduce relapse rates and contribute to the development of effective intervention plans. In the present study, we attempted to explore the personality traits that could be associated with the return of depression while considering important co-factors (i.e., clinical and social) on, to some extent, an international sample. This study aimed to investigate the association between the return of Major Depressive Disorder and personality traits of emotional dysregulation, affective lability, and impulsivity. It is hypothesised that there will be a significant relationship between emotional dysregulation, affective lability, impulsivity, and the return of depression (relapse and recurrence).

## 2 Methods

This study obtained ethical approval from the University of Birmingham’s Science, Technology, Engineering and Mathematics Ethics Review Committee.

### 2.1 Study design

A case–control design consisting of individuals with a history of depression and currently either experiencing a depressive episode or in remission. According to the Patient Health Questionnaire-9 (PHQ-9) (Kroenke et al., 2001), the sample was divided into two groups: cases, who were participants with a current major depressive episode; controls were participants with no current major depressive episode.

### 2.2 Participants

Respondents were adults (18 years and over) with a history of depression who were recruited online. The data was collected using (Qualtrics) which is a secure survey software; more details can be found in the recruitment procedure section of this paper.

The inclusion criteria were participants aged 18 years or over who were previously diagnosed and treated for major depression over their lifetime and recovered according to their self-report. The exclusion criteria were participants under the age of 18 or with no history of MDD. Eligible participants were identified using two questions: Have you ever been diagnosed with and treated for depression and recovered? The second question was: Are you over the age of 18? Participants were considered eligible if they answered YES to both questions.

### 2.3 Study measures

All study measures were delivered to participants online. We stress that the cross-sectional design involved in the present study limits the use of clinical terms such as relapse and recurrence accurately. Therefore, in this paper, the authors will be referring to this phenomenon by the term (return of depression), to describe a current major depressive episode after a period of recovery reported by a participant in a self-report questionnaire.

*Current major depressive episode*: MDD was assessed using The Patient Health Questionnaire-9 (PHQ-9) (Kroenke et al., 2001), a widely used self-report tool to assess depression in medical settings. PHQ-9 showed good validity and sensitivity (0.83) in measuring MDD symptoms and their severity in a large psychiatric sample (*n* = 1,023) (Beard et al., 2016). The present study assessed the internal consistency of this scale using Cronbach’s *α*. The survey’s Cronbach’s Alpha value was *α* = 0.87, indicating good reliability. The PHQ-9 comprises nine items that score from 0 to 27; a cut-score of ≥10 indicates a current major depressive episode and was used in this study to indicate current MDD.
*Emotional dysregulation*: Emotional dysregulation was measured using the short form of the Difficulties in Emotion Regulation Scale (DERS-16) (Bjureberg et al., 2016). DERS-16 is a self-report questionnaire designed to assess several elements of emotional dysregulation. It comprises 16 items; after reading each statement, respondents were asked to select the appropriate number on a scale from 1 (almost never) to 5 (almost always) to indicate how often it is applied to them. Higher scores indicate greater emotion regulation difficulties. The measure produce a total score (SUM) I addition to scores on five sub-scales: non-acceptance of emotional responses (NONACCEPT), difficulties engaging in goal-directed behaviour (GOALS), impulse control difficulties (IMPULSE), limited access to emotion regulation strategies (STRATEGIES), and lack of emotional clarity (CLARITY). Each DERS subscales demonstrated sufficient internal consistency, with Cronbach’s *α* >0.70 for every subscale (Burton et al., 2022). In the present study, we assessed the internal consistency of this scale using Cronbach’s *α*. The survey’s Cronbach’s Alpha value was *α* = 0.94, indicating excellent reliability. The total score (SUM) was used as a continuous variable in the analysis phase.
*The affective lability scale - short form (ALS-18)*: The 18-item version comprises 18 items representing three subscales: anxiety/depression shifts (5 items), depression/elation shifts (8 items), and anger (5 items) (Oliver and Simons, 2004). The mean scores for each item are calculated to extract the overall score. The current study assessed the internal consistency of this scale using Cronbach’s α. The survey’s Cronbach’s Alpha value was *α* = 0.93, indicating excellent reliability. The total score (SUM) was used as a continuous variable in the analysis phase.
*Impulsivity*: Impulsivity was measured using the Barratt Impulsiveness Scale (BIS-11) (Patton et al., 1995), the most commonly used instrument for assessing impulsivity. The BIS-11 was built to measure impulsivity represented by three main aspects: attentional (attention and cognitive instability), motor (motor and perseverance), and non-planning (self-control and cognitive complexity). It consists of 30 statements that indicate common impulsive or non-impulsive (in the case of reverse-scoring items) behaviours and preferences. Items are scored on a 4-point scale (Rarely/Never = 1, Occasionally = 2, Often = 3, Almost Always/Always = 4). Higher overall scores, which range from 30 to 120, indicate greater impulsivity. The current study assessed the internal consistency of this scale using Cronbach’s *α*. The survey’s Cronbach’s Alpha value was α = 0.62, indicating acceptable reliability. The total score (SUM) was used as a continuous variable in the analysis phase.

### 2.4 Covariates

In addition to the main variables of this study, we were interested in assessing further co-variables that have a potential impact on the return of MDD so that we observe their effect through the statistical analysis phase. These factors were measured as follows:

*Current treatment for depression*: Participants were asked (Are you currently undergoing any treatment for depression? (yes/ no)) and (Did you stop treatment for depression in the last 3 months? (yes/ no)).
*The number of previous depressive episodes*: Participants were asked (How many depressive episodes did you experience in the past?). Participants should select an option from a drop-down list of (one episode, two episodes, three episodes or more; I am not sure), coded as 0, 1, 2 and 3, respectively.
*Generalised anxiety disorder*: (GAD) was measured using the GAD-7 (Spitzer et al., 2006), a brief self-report scale. It aims to assess an individual’s anxiety level by assigning scores from 0 to 3 for each item, where 0 = not at all, 1 = several days, 2 = more than half the days, and 3 = nearly every day. Total score ranges from 0 to 21; this can fall under one of four categories: 0–4: minimal anxiety, 5–9: mild anxiety, 10–14: moderate anxiety, and 15–21: severe anxiety. The current study assessed the internal consistency of this scale using Cronbach’s *α*. The survey’s Cronbach’s Alpha value was α = 0.90, indicating excellent reliability.
*Childhood trauma*: This factor was measured using the Childhood Trauma Questionnaire-Short Form (CTQ) (Bernstein et al., 2003). It is a self-report retrospective tool designed to assess different aspects of childhood abuse. This scale consists of 28 items represented in five sub-scales: Physical abuse, sexual abuse, emotional abuse, physical neglect, and emotional neglect. Items are scored on a 5-point Likert scale: (1 = never true, 2 = rarely true, 3 = sometimes true, 4 = often true, and 5 = very often true). Additionally, this scale consists of an additional scale of three questions (minimization/denial scale) to assess the likelihood of unreported traumatic experiences. The current study assessed the internal consistency of this scale using Cronbach’s *α*. The survey’s Cronbach’s Alpha value was α = 0.71, indicating acceptable reliability. The total score (SUM) was used as a continuous variable in the analysis phase.
*Personality disorders history*: Participants were asked (Have you ever been diagnosed with a personality disorder?), yes or no.
*Treatment status for physical health*: They were also asked (please indicate if you are currently being treated for any of the following conditions). Participants should select an option from a drop-down list of (substance use problems, cancer, diabetes, chronic pain, HIV, a thyroid condition, or another condition).

All participants were provided with a consent form alongside a demographic questionnaire. A diagram of the order of the study questionnaires is available in a Supplementary Data Sheet 1.

### 2.5 Recruitment procedure

The study was advertised through the student/ staff email list at the University of Birmingham and the official Twitter accounts of the institutions the authors belong to in the UK (see diagram 1 in the Supplementary Data Sheet 1). In addition, a link was created that led to the study measures being administered electronically to potential participants. A unique number was given to each participant automatically to allow the identification of each participant. The identification using a unique number allowed us to reach and discard data belonging to participants who wished to withdraw from the study. Participants remained anonymous even with the unique number allocated, as no questions could identify their identities, such as their names, date of birth, or address.

### 2.6 Statistical analysis plan

SPSS version (29.0.0.0) was used to conduct the statistical analysis for this study. Descriptive statistics that describe the basic features of the data were used to investigate participants’ demographics and their characteristics on personality traits, childhood trauma, and clinical factors in both groups. First, a chi-square test of independence was performed to assess the association between the sociodemographic variables and other relevant questions on participants’ health and the return of MDD. Fisher’s exact test was applied to overcome limitations in cells that included a number of participants less than five. An independent sample t-test was used to measure the differences between groups on the personality and psychopathology variables. Second, a univariate logistic regression was utilised to assess the association between the explanatory variables and the return of MDD. The next step was performing a multiple logistic regression to investigate the association between the return of MDD and psychopathology factors that were significant in the univariate model. Given the high correlation between emotional dysregulation and affective lability, we decided to assess the association between psychopathology and personality factors and the return of depression using three models. In the first model, both emotional dysregulation and AL were entered alongside the psychopathology factors. From personality factors, only emotional dysregulation was included in the second model and only AL was included in the third one. This approach allowed us to explore the effect of emotional dysregulation and affective lability on the return of MDD independently. Finally, an adjusted multiple logistic regression was conducted to control the confounding factors that showed a significant association with the return of MDD in the univariate analysis.

In relation to missing data, we performed propensity score weighting (Olmos and Govindasamy, 2019). We developed a logistic regression model (non-response vs. response outcome) and entered the main covariates of our study to calculate the predicted probability of responding to our survey based on the covariates we included in this model. Next, we created a new variable called Weight based on the predicted probability to determine weights for each respondent to account for non-participants. We activated the (weighting cases) function in SPSS during the analysis. Results in this study were considered significant when *p* values were < 0.05.

### 2.7 Power and sample size estimation

This study aimed to test the differences between individuals who experienced a return of MDD (cases) and individuals with no return of MDD (controls) on emotional dysregulation scores. Then, the association between emotional dysregulation and the return of MDD was evaluated. Based on previous studies on emotional dysregulation and depression (Ehring et al., 2008), we estimated a small to medium effect size (*d* = 0.46). The estimated sample size necessary to determine a small to medium effect size at an alpha rate of 0.05 (two-tailed) using the G*Power Programme was 152 respondents (76 cases and 76 controls).

## 3 Results

Initially, 1,032 participants (*N* = 1,032) accessed the link online to the study. Of those, 290 (*N* = 290) were eligible to participate according to the study criteria. 138 participants were excluded due to failing to respond to over 60% of the study questionnaires. 152 participants (*N* = 152) were included in the current analysis (Males 41, females 108, other 3), of which 76 were reported to have current depression, according to the PHQ-9, representing cases, whereas 76 did not report current depression, according to the same scale, representing controls. Both groups self-reported a history of major depression. Characteristics of the sample on the study variables are shown in Table 1.

**Table 1:** General characteristics of study (*n* = 152).

Characteristics	Return of MDD *n* = 76 (50%)	No return of MDD *n* = 76 (50%)	*P*-value
Sex, *n* (%)			
Male	21 (27.63%)	20 (26.3%)	0.264
Female	52 (68.42%)	56 (73.7%)	
Other	3 (3.94%)	0 (0%)	
Age, mean (SD)	29.55 (7.12)	30.43 (6.84)	500
Ethnicity, *n* (%)			
Arab	60 (78.94%)	57 (75%)	0.452
White	11 (14.47%)	15 (19.7%)	
Other	5 (6.57%)	4 (5.26%)	
Marital status, *n* (%)			
Married	17 (22.36%)	27 (35.5%)	**0.014**
Widowed/Divorced/Separated	7 (9.21%)	1 (1.3%)	
Single	52 (68.42%)	48 (63.2%)	
Employment, *n* (%)			
Working full-time	41 (53.94%)	46 (60.5%)	0.263
Working part-time	5 (6.57%)	4 (5.26%)	
Unemployed	26 (34.21%)	18 (23.7%)	
Economically inactive	4 (5.26%)	8 (10.5%)	
Number of previous depressive episodes, *n* (%)			
One	4 (5.26%)	11 (14.5%)	0.071
Two	7 (9.21%)	11 (14.5%)	
Three or more	34 (44.73%)	30 (39.5%)	
I am not sure	31 (40.78%)	24 (31.6%)	
Current treatment for depression, *n* (%)			
Psychological treatment	6 (7.89)	5 (6.58)	0.491
Antidepressants	14 (18.42)	14 (18.42)	
Both	13 (17.10)	6 (7.89)	
Other	2 (2.63)	2 (2.63)	
I am not undergoing any treatment for depression	41 (53.94)	49 (64.47)	
Did you stop treatment for depression in the last 3 months? *n* (%)			
Yes	33 (43.42%)	18 (23.68%)	**0.006**
No	43 (56.57%)	58 (76.31%)	
Other current mental health problems, *n* (%)			
Yes	22 (28.94%)	15 (19.7%)	0.205
No	54 (71.05%)	61 (80.3%)	
History of personality disorders, *n* (%)			
Yes	18 (23.68%)	6 (7.9%)	**0.008**
No	58 (76.31%)	70 (92.1%)	
Current treatment for drug and/or alcohol dependency, *n* (%)			
Yes	4 (5.26%)	0 (0%)	0.121
No	72 (94.73%)	76 (100%)	
Current treatment for psychosis, *n* (%)			
Yes	2 (2.63%)	2 (2.6%)	1.00
No	74 (97.36%)	74 (97.4%)	
Current physical health condition, *n* (%)			
Yes	17 (22.36%)	13 (17.10%)	0.705
No	59 (77.63%)	63 (82.9%)	
GAD, *n* (%)			
Mild	16 (21.05%)	36 (47.36%)	**<0.001**
Moderate	20 (26.31%)	15 (19.73%)	
Severe	35 (46.05%)	9 (11.84%)	
No GAD	5 (6.57%)	16 (21.05%)	
DERS total score, mean (SD)	54.79 (13.74)	41.11 (15.06)	**<0.001**
ALS total score, mean (SD)	30.11 (12.46)	24.45 (13.008)	**0.005**
BIS total score, mean (SD)	68.87 (8.96)	67.37 (7.33)	0.306
CTQ total score, mean (SD)	59.46 (12.48)	54.01 (9.30)	**0.002**

SD, standard deviation. Significant figures are shown in bold fonts. GAD, Generalized Anxiety Disorder. DERS, Difficulties in Emotion Regulation Scale. ALS, Affective Lability Scale. BIS, Barratt Impulsiveness Scale. CTQ, Childhood Trauma Questionnaire.

Fisher’s exact test showed that among all sociodemographic factors, marital status was the only factor that was significantly associated with the return of MDD (two-tailed *p* = 0.014) (Table 1).

There was a statistically significant relationship between the history of personality disorders and the reported return of depression; *X*^2^ ([1], *N* = [152]) = [7.75], *P* = [0.008]. The same test also showed that generalised anxiety disorder was strongly associated with the return of depression; *X*^2^ ([3], *N* = [152]) = [31.62], *P* = [<0.001]. The groups also differ significantly on the question (Did you stop treatment for depression in the last three months?) *X*^2^ ([1], *N* = [152]) = [8.18], *P* = [0.006] (Table 1).

About personality traits, an independent two samples t-test revealed significant differences in DERS scores between [cases] (*M* = [54.68], SD = [13.90]) and [controls] (*M* = [41.07], SD = [15.10]); t(167) = [−6.1], *p* = [<0.001]. The two groups also differed in ALS scores, [cases] (*M* = [29.91], SD = [12.51]) and [controls] (*M* = [24.38], SD = [13.03]); t(167) = [−2.81], *p* = [0.005]. The test did not show significant differences between cases and controls in the BIS scores, [cases] (*M* = [68.77], SD = [8.99]) and [controls] (*M* = [67.47], SD = [7.47]); t(167) = [−1.03], *p* = [0.306] (Table 1).

Furthermore, our analysis revealed that there was a significant difference between groups in the CTQ scores, according to an independent two samples t-test, [cases] (*M* = [59.17], SD = [12.49]) and [controls] (*M* = [53.90], SD = [9.39]); t(167) = [−3.09], *p* = [0.002].

Second, a univariate logistic regression revealed that all variables significantly associated with the return of depression in the first analysis remained significant in this model. See Table 2 for details.

**Table 2:** Univariate logistic regression of association between the study variables and the return of depression (*n* = 152).

Factors	OR (95%CI)	*P-*value
Marital status		
Married	Ref	
Widowed/Divorced/Separated	9.37 (1.33–65.66)	**0.024**
Single	1.79 (0.90–3.56)	0.097
History of personality disorders		
Yes	3.58 (1.37–9.31)	**0.009**
No	Ref	Ref
GAD		
Mild	1.36 (0.46–4.00)	0.570
Moderate	4.06 (1.32–12.48)	**0.014**
Severe	11.36 (3.58–36)	**<0.001**
No GAD	Ref	Ref
Did you stop treatment for depression in the last 3 months? *n* (%)		
Yes	2.56 (1.32–4.97)	**0.005**
No	Ref	Ref
DERS total score	1.06 (1.03–1.08)	**<0.001**
ALS total score	1.03 (1–1.06)	**0.007**
CTQ total score	1.04 (1.01–1.08)	**0.004**

The dependent variable in this analysis is the return of MDD coded so that 0 = no return of MDD and 1 = return of MDD. GAD, Generalized Anxiety Disorder. ALS, Affective Lability Scale. CTQ, Childhood Trauma Questionnaire. Significant figures are shown in bold font.

Third, we were interested in exploring the association between personality traits alongside psychopathology factors and the return of depression; therefore, a multiple logistic regression was utilised. Pearson correlation test between emotional dysregulation and AL revealed that there is a significant, strong positive relationship between the two personality constructs, *r* [(167]) = [0.50], *p* = [<0.001]. Accordingly, three models of multiple logistic regression were conducted to assess the impact of the two personality factors on the return of depression independently. In model A, where both emotional dysregulation and AL were entered, results showed that emotional dysregulation was significantly associated with the return of depression. An increase of one score on the DERS scale increases the odds of experiencing a return of depression by 1.04 times (OR = 1.04, 95% CI [1.01–1.07]). Severe anxiety was another factor that showed a significant association with the return of depression, according to this model. Results showed that the odds ratio for individuals with severe anxiety is 4.28, with a 95% confidence interval of [1.10–16.69]. No other explanatory variables appear to be associated with the return of depression in this model (Table 3).

**Table 3:** Multiple logistic regression of association between the psychopathology factors and the return of depression (*n* = 152), model A.

Factors	OR (95%CI)	*P*-value
DERS total score	1.04 (1.01–1.07)	**0.004**
ALS total score	0.989 (0.95–1.02)	0.537
GAD		
Mild	0.802 (0.24–2.65)	0.717
Moderate	1.77 (0.49–6.34)	0.380
Severe	4.28 (1.10–16.69)	**0.036**
No GAD	Ref	Ref
History of personality disorders		
Yes	2.22 (0.75–6.58)	0.151
No	Ref	Ref
Did you stop treatment for depression in the last 3 months? *n* (%)		
Yes	1.93 (0.89–4.15)	0.093
No	Ref	Ref

The dependent variable in this analysis is the return of MDD coded so that 0 = no return of MDD and 1 = return of MDD. DERS, Difficulties in Emotion Regulation Scale. ALS, Affective Lability Scale. GAD, Generalized Anxiety Disorder. Significant figures are shown in bold font.

We found the same findings from model A in model B (emotional dysregulation included), in which only high DERS scores and severe GAD were significantly associated with the return of MDD (OR = 1.04, 95% CI [1.01–1.07]), (OR = 3.90, 95% CI [1.03–14.72]), respectively. See Table 4. In model C (AL included), the analysis did not detect a relationship between ALS scores and the return of MDD. Moderate to severe GAD appeared to be associated with the return of depression in this model. See Table 5 for details.

**Table 4:** Multiple logistic regression of association between the psychopathology factors and the return of depression (*n* = 152), model B.

Factors	OR (95%CI)	*P*-value
DERS total score	1.04 (1.01–1.07)	**0.003**
GAD		
Mild	0.764 (0.23–2.50)	0.657
Moderate	1.69 (0.47–6.0)	0.419
Severe	3.90 (1.03–14.72)	**0.004**
No GAD	Ref	Ref
History of personality disorders		
Yes	2.14 (0.73–6.27)	0.163
No	Ref	Ref
Did you stop treatment for depression in the last 3 months? *n* (%)		
Yes	1.94 (0.90–4.17)	0.088
No	Ref	Ref

The dependent variable in this analysis is the return of MDD coded so that 0 = no return of MDD and 1 = return of MDD. DERS, Difficulties in Emotion Regulation Scale. GAD, Generalized Anxiety Disorder. Significant figures are shown in bold font.

**Table 5:** Multiple logistic regression of association between the psychopathology factors and the return of depression (*n* = 152), model C.

Factors	OR (95%CI)	*P*-value
ALS total score	1.00 (0.98–1.04)	0.551
GAD		
Mild	1.11 (0.35–3.45)	0.854
Moderate	3.15 (0.96–10.35)	**0.058**
Severe	8.33 (2.37–29.30)	**<0.001**
No GAD	Ref	Ref
History of personality disorders		
Yes	2.59 (0.90–7.41)	0.076
No	Ref	Ref
Did you stop treatment for depression in the last 3 months? *n* (%)		
Yes	2.06 (0.98–4.32)	**0.056**
No	Ref	Ref

The dependent variable in this analysis is the return of MDD coded so that 0 = no return of MDD and 1 = return of MDD. ALS, Affective Lability Scale. GAD, Generalized Anxiety Disorder. Significant figures are shown in bold font.

Finally, we conducted a multiple logistic regression, this time with control of confounding factors significantly associated with the return of depression in the first analysis (i.e., marital status and CTQ scores); results are displayed in Table 6. Again, results showed that emotional dysregulation and severe GAD were robust factors that remained significant in all models in this study. See Table 6 for details. This model also revealed that widowed, divorced, and separated respondents were more likely to experience a return of depression compared to married ones. The odds ratio for individuals who are widowed, divorced, or separated is 13.95, with a 95% confidence interval of [1.16–166]. Results also showed that childhood trauma was significantly associated with the return of depression. An increase of one score on the CTQ scale increases the odds of experiencing a return of depression by 1.04 times (OR = 1.04, 95% CI [1.00–1.08]) (Table 6).

**Table 6:** Multiple logistic regression of association between the psychopathology factors and the return of depression with the control of confounding factors (*n* = 152).

Factors	OR (95%CI)	*P*-value
DERS total score	1.03 (1.00–1.06)	**0.040**
ALS total score	0.987 (0.95–1.0)	0.454
GAD		
Mild	1.16 (0.33–4.08)	0.812
Moderate	2.49 (0.65–9.60)	0.184
Severe	6.29 (1.49–26.58)	**0.012**
No GAD	Ref	Ref
History of personality disorders		
Yes	2.23 (0.72–6.88)	0.160
No	Ref	Ref
Did you stop treatment for depression in the last 3 months? *n* (%)		
Yes	1.93 (0.87–4.31)	0.104
No	Ref	Ref
Marital status		
Married	Ref	Ref
Widowed/Divorced/Separated	13.95 (1.16–166)	**0.037**
Single	2.13 (0.90–4.99)	0.082
CTQ total score	1.04 (1.00–1.08)	**0.034**

The dependent variable in this analysis is the return of MDD coded so that 0 = no return of MDD and 1 = return of MDD. DERS, Difficulties in Emotion Regulation Scale. ALS, Affective Lability Scale. GAD, Generalized Anxiety Disorder. CTQ, Childhood Trauma Questionnaire. Significant figures are shown in bold font.

## 4 Discussion

We aimed to investigate the association between the return of MDD and emotional dysregulation, affective lability, and impulsivity. The primary outcome was emotional dysregulation. This was defined in this study as a current greater score on the short form of the Difficulties in Emotion Regulation Scale (DERS-16) (Bjureberg et al., 2016), which reflects higher levels of emotional dysregulation. Our findings showed that individuals with higher levels of emotional dysregulation, GAD and childhood trauma are more prone to the return of depression compared to individuals without these conditions. This suggests that emotional dysregulation and childhood trauma could work as risk factors and predate depression. We also found that widowed, divorced and separated respondents could be more vulnerable to a return of depression compared to married ones. We did not find significant differences in affective lability and impulsivity scores between cases and controls.

Our results on the association between emotional dysregulation and depression are in keeping with previous research (Liu and Thompson, 2017; Visted et al., 2018). One possible explanation of the relationship between emotional dysregulation and depression is that increased rumination, expressive suppression, and impaired reappraisal are associated with emotional dysregulation. This, in turn, is related to an impairment in processing unpleasant materials, a core element of MDD (Compare et al., 2014). On a cognitive level, a review by LeMoult and Gotlib (2019) revealed that research has increasingly documented the significant role of cognitive emotional dysregulation strategies (e.g., rumination and reappraisal) in depression. Increased rumination and decreased reappraisal were found to be common characteristics in people with depression (LeMoult and Gotlib, 2019). Further, difficulties in emotional regulation and the lack of adaptive emotional regulation strategies in adolescence were found to predict depressive symptoms 2 years later (Gonçalves et al., 2019). This highlights the importance of early efforts in depression prevention.

Regardless of the terms used in describing difficulties in regulating emotions, research has consistently demonstrated the role that negative emotionality and intense fluctuating mood play in understanding depression aetiology (Mennin et al., 2007). For example, mood instability MI was found to be strongly correlated with depressed people compared to those with no depression (Bowen et al., 2017). MI is also a fundamental component in neuroticism (Bowen et al., 2012), which is widely documented to be a core feature of depression (Navrady et al., 2017). Altered emotions have frequently been reported to be associated with mood psychopathology. A prospective study on emotion dynamics by Sperry et al. (2020) found that negative emotion instability predicted depression at 3 years follow-up. The relationship between unstable mood and depressive psychopathology could be partly explained by the notion that individuals with intense, frequent, fluctuating moods are more likely to generate stressful life events, which may lead to depression. For instance, a study by Miller and Pilkonis (2006) revealed that affective instability predicted interpersonal impairment (i.e., romantic relationships), and such events could contribute to the development of depression.

The finding that GAD is one of the most robust factors associated with the return of depression highlights the complex and intertwined nature of these two mental health conditions. Anxiety and depression are often comorbid, with lifetime co-occurrence rates reported to exceed 50% in some studies (Kessler et al., 2015). This high degree of overlap has prompted significant research into the mechanisms underpinning their association.

Cognitive and behavioural factors provide important insights into the association between GAD and the return of depression. Anxiety often manifests as excessive worry and heightened threat sensitivity, which can amplify cognitive distortions commonly associated with depression, such as negative self-appraisals and hopelessness (Beck and Barlow, 2017). This cognitive overlap may create a self-reinforcing cycle, wherein anxiety exacerbates depressive symptoms and vice versa, increasing the likelihood of relapse or recurrence of depression.

From a psychosocial perspective, individuals with GAD may encounter chronic stressors, reduced social support, and impaired functioning, all of which are established risk factors for depressive episodes (Rudolph et al., 2014). Additionally, persistent anxiety symptoms may erode coping resources, leaving individuals more vulnerable to stress-induced depressive relapses. For instance, longitudinal research has demonstrated that individuals with comorbid anxiety and depression experience more severe and persistent symptoms compared to those with depression alone (Kendler et al., 2003).

It is also important to consider the role of personality traits and disorders in this association. High levels of neuroticism, which have been linked to both GAD and depression, may act as a shared diathesis that predisposes individuals to emotional dysregulation and recurrent depressive episodes (Lahey, 2009). Furthermore, as evidenced in our previous research, borderline personality traits can exacerbate the course of depression, potentially influencing the relationship between anxiety and depressive relapse (Altaweel et al., 2024).

Our findings also showed that childhood trauma is a possible risk factor for the return of depression. The fact that childhood trauma and emotional dysregulation are very linked has been supported in the literature, and possible mechanisms of this link were presented in several research. An example of this includes a review by Teicher et al. (2016), which investigated the impact of childhood maltreatment on brain structure and functions. The review revealed that childhood maltreatment, particularly physical and emotional abuse and neglect, has a substantial influence on the development of significant emotion centres in the brain (ie., amygdala and hippocampus), considered a critical risk factor for adult psychopathology (Teicher et al., 2016). Furthermore, emotional regulation difficulties were found to mediate the relationship between depression and childhood trauma in patients with MDD (Hopfinger et al., 2016; Huh et al., 2017).

Contrary to expectations, this study did not find a significant difference between cases and controls on affective lability scores. This result differs from studies of Zwicker et al. (2020) and Høegh et al. (2022), where affective liability was linked to mental disorders, including depression. The high statistical correlation reported in the present paper between emotional dysregulation and AL could partly explain our finding as they are overlapping constructs.

Social impairments are a critical component that is frequently reported in the literature to be affected by affective dysregulation generally and developing depression (e.g., Høegh et al., 2022). The available evidence suggests that (e.g., affective lability) significantly and negatively affects the social functioning of people with severe mental disorders (Høegh et al., 2022). Therefore, it might be worthwhile to understand the relationship between emotional regulation difficulties in all its forms and depression by looking at the quality of social functioning.

Although some studies have reported a relationship between impulsivity and remitted depression (Saddichha and Schuetz, 2014), our findings did not identify a statistically significant relationship between impulsivity and the return of MDD. It is challenging to assume a direct relationship between impulsivity and depression, as the available research in this area presents conflicting findings (Fields et al., 2021; Ngo et al., 2011).

This inconsistency is likely due to the multidimensional nature of impulsivity, which encompasses various facets, including lack of premeditation, urgency, sensation-seeking, and lack of perseverance (Whiteside and Lynam, 2001). Results often depend on the specific facet being assessed and the type of assessment used (Fields et al., 2021). In our study, we used a self-report measure to assess impulsivity, which, while well-suited for capturing trait impulsivity, may not fully reflect situational or behavioural dimensions that could be more closely linked to depression outcomes (Cyders and Coskunpinar, 2011). Self-report tools often aggregate multiple facets of impulsivity, potentially obscuring the specific contributions of individual dimensions, such as urgency or lack of premeditation, to depression relapse or recurrence (Berg et al., 2015).

Furthermore, much of the existing research on impulsivity and depression has focused on its role in the context of suicidality (Ekinci et al., 2011). While our study did not exclude participants with suicidality, the lack of suicidality-specific analyses limits our understanding of how impulsivity operates in its absence. This highlights the need for further investigation into the causal mechanisms and nuanced dynamics between impulsivity and depression when suicidality is not a confounding factor.

Finally, the heterogeneity in study designs and population characteristics may contribute to the inconsistent findings in the literature. Impulsivity may play distinct roles in depression onset, relapse, and recurrence (Hershenberg et al., 2020). Our study utilised a cross-sectional case–control design, which provides a snapshot of group differences but does not capture temporal or situational changes in impulsivity. This limitation could partially explain the absence of a significant association in our findings.

The results of the present study provide further support for the critical contribution of emotional dysregulation in the return of depression. An implication of this is the possibility of developing effective interventions that target emotional dysfunctions that could help reduce relapse rates after an individual has recovered from an acute episode of depression. On the individual level, current findings offer insights into the importance of emotional regulation in managing favourable mental health.

Finally, the current study was limited by first, relying on self-report questionnaires, including measuring MDD, which ideally needs a clinical assessment alongside the scales to indicate MDD diagnosis. Second, performing a cross-sectional methodological design where a prospective approach could be ideal in examining long-term depression outcomes. This design could not allow the use of accurate clinical terms such as relapse or recurrence as we relied on participants reporting they had a history of depression and recovered. Third, the relatively small final sample size (*N* = 152) compared to the total number of individuals who initially accessed the study link (*N* = 1,032) was another limitation. The final sample size was a result of applying eligibility criteria and excluding participants who provided incomplete responses. While these measures were essential to ensure data quality and adherence to the study’s objectives, they reduced the representativeness of the sample. This limitation should be considered when interpreting the findings, and future research may benefit from strategies to maximise participant retention and minimise data loss.

Fourth, the demographic profile of the study participants, particularly the young Arab women, is striking and warrants further discussion regarding the recruitment process. This notable demographic trend may reflect cultural factors and the use of social media platforms as a primary recruitment tool. Given its accessibility and perceived anonymity, social media often attracts younger audiences and allows Arab women to engage more openly with academic and health-related initiatives. The online survey recruitment strategy may have unintentionally overrepresented this demographic, as internet use among younger individuals in Arab countries is higher than among older populations (Barometer, n.d.; International Telecommunication Union [ITU], 2023). Therefore, this study’s results should be interpreted with caution, considering that the findings may be more reflective of the experiences of young Arab women than of the wider population.

Fifth, a notable limitation of this study is the dropout rate observed during questionnaire completion. This may have resulted from factors such as the perceived length of the survey, the sensitivity of the topics addressed, or situational and technical interruptions faced by participants. Future studies should consider employing shorter questionnaires, culturally sensitive designs, and adaptive methods to improve participant retention.

Finally, we acknowledge the limitation of relying solely on self-report questionnaires to rule out the presence of personality disorders. This approach may introduce some uncertainty, as self-report measures are less comprehensive than structured clinical interviews and may not detect all cases of personality disorder. While this potential misclassification could influence the findings, self-report instruments remain a widely accepted and practical method in large-scale studies for assessing personality traits and psychopathology.

Notwithstanding these limitations, the strengths of our study manifested in involving respondents from diverse ethnic groups. In addition, the study variables were measured using internationally recognised scales. The data analysis plan also included several models to accurately explore the association between the risk factors and the outcome, controlling for important confounding factors reported in the literature to associate with the return of depression.

## 5 Conclusion

The present study aimed to examine the role of personality traits of emotional dysregulation, affective lability and impulsivity in the return of MDD. We found that higher emotional dysregulation and severe GAD were the most robust risk factors for the return of MDD. It was also shown that individuals who reported childhood trauma and individuals who are widowed, divorced or separated are more vulnerable to a return of depression. Our analysis did not detect any association between affective lability and impulsivity and the return of MDD. This information can be used to develop targeted treatment plans that consider improving depressed patients’ skills in managing their emotions as one way to fight the depressive relapse phenomenon. Future work could address this issue using a longitudinal approach, examining potential mediators between personality traits and the return of MDD.

## Data Availability

The raw data supporting the conclusions of this article will be made available by the authors, without undue reservation.

## References

[ref9001] Altaweel N. Upthegrove R. Marwaha S. (2024). Personality traits and change in depression status at 18 months: Findings from a British Psychiatric Morbidity Survey. J. Affect. Disord. 367, 157–163. 39222851 10.1016/j.jad.2024.08.190

[ref1] Altaweel N. Upthegrove R. Surtees A. Durdurak B. Marwaha S. (2023). Personality traits as risk factors for relapse or recurrence in major depression: a systematic review. Front. Psych. 14:709. doi: 10.3389/fpsyt.2023.1176355, PMID: 37215669 PMC10196019

[ref9002] Barnhofer T. Chittka T. (2010). Cognitive reactivity mediates the relationship between neuroticism and depression. Behav. Res. Ther. 48, 275–281. 20070952 10.1016/j.brat.2009.12.005 PMC2850390

[ref3] Barometer Arab. (n.d.). Predictors of internet usage in the Arab world. Avaialable online at: https://www.arabbarometer.org (Accessed November 16, 2024).

[ref4] Beard C. Hsu K. J. Rifkin L. S. Busch A. B. Björgvinsson T. (2016). Validation of the PHQ-9 in a psychiatric sample. J. Affect. Disord. 193, 267–273. doi: 10.1016/j.jad.2015.12.075, PMID: 26774513

[ref9003] Beck A. T. Barlow D. H. (2017). Cognitive approaches to anxiety and depression: The integrative perspective. J. Clin. Psychol. 73, 929–940. doi: 10.1002/jclp.22473 28675782

[ref5] Berg J. M. Latzman R. D. Bliwise N. G. Lilienfeld S. O. (2015). Parsing the heterogeneity of impulsivity: a meta-analytic review of the behavioural implications of the UPPS for psychopathology. Psychol. Assess. 27, 1129–1146. doi: 10.1037/pas0000111, PMID: 25822833

[ref6] Bernstein D. P. Stein J. A. Newcomb M. D. Walker E. Pogge D. Ahluvalia T.. (2003). Development and validation of a brief screening version of the childhood trauma questionnaire. Child Abuse Negl. 27, 169–190. doi: 10.1016/S0145-2134(02)00541-0, PMID: 12615092

[ref7] Bjureberg J. Ljótsson B. Tull M. T. Hedman E. Sahlin H. Lundh L. G.. (2016). Development and validation of a brief version of the difficulties in emotion regulation scale: the DERS-16. J. Psychopathol. Behav. Assess. 38, 284–296. doi: 10.1007/s10862-015-9514-x, PMID: 27239096 PMC4882111

[ref8] Bowen R. Balbuena L. Leuschen C. Baetz M. (2012). Mood instability is the distinctive feature of neuroticism. Results from the British health and lifestyle study (HALS). Personal. Individ. Differ. 53, 896–900. doi: 10.1016/j.paid.2012.07.003

[ref9] Bowen R. Peters E. Marwaha S. Baetz M. Balbuena L. (2017). Moods in clinical depression are more unstable than severe normal sadness. Front. Psych. 8:56. doi: 10.3389/fpsyt.2017.00056, PMID: 28446884 PMC5388683

[ref10] Buckman J. E. Underwood A. Clarke K.. (2018). Risk factors for relapse and recurrence of depression in adults and how they operate: a four-phase systematic review and meta-synthesis. Clin. Psychol. Rev. 64, 13–38. doi: 10.1016/j.cpr.2018.07.005, PMID: 30075313 PMC6237833

[ref11] Burcusa S. L. Iacono W. G. (2007). Risk for recurrence in depression. Clin. Psychol. Rev. 27, 959–985. doi: 10.1016/j.cpr.2007.02.005 17448579 PMC2169519

[ref12] Burton A. L. Brown R. Abbott M. J. (2022). Overcoming difficulties in measuring emotional regulation: assessing and comparing the psychometric properties of the DERS long and short forms. Cogent Psychol. 9:2060629. doi: 10.1080/23311908.2022.2060629

[ref9004] Clark L. A. Fisher A. J. Zimmerman J. (2020). On the interpersonal and emotional core of personality pathology. Pers. Disord.: Theory Res. Treat. 11, 70–80.

[ref13] Compare A. Zarbo C. Shonin E. van Gordon W. Marconi C. (2014). Emotional regulation and depression: a potential mediator between heart and mind. Cardiovasc. Psychiatry Neurol. 2014, 1–10. doi: 10.1155/2014/324374 PMC4090567 25050177

[ref14] Corruble E. Benyamina A. Bayle F. Falissard B. Hardy P. (2003). Understanding impulsivity in severe depression? A psychometrical contribution. Prog. Neuro-Psychopharmacol. Biol. Psychiatry 27, 829–833. doi: 10.1016/S0278-5846(03)00115-5, PMID: 12921916

[ref15] Cyders M. A. Coskunpinar A. (2011). Measurement of constructs using self-report and behavioural lab tasks: is there overlap in nomothetic span and construct representation for impulsivity? Clin. Psychol. Rev. 31, 965–982. doi: 10.1016/j.cpr.2011.06.001, PMID: 21733491

[ref16] Dalley J. W. Everitt B. J. Robbins T. W. (2011). Impulsivity, compulsivity, and top-down cognitive control. Neuron 69, 680–694. doi: 10.1016/j.neuron.2011.01.020 21338879

[ref17] Ehring T. Fischer S. Schnulle J.. (2008). Characteristics of emotion regulation in recovered depressed versus never depressed individuals. Personal. Individ. Differ. 44, 1574–1584. doi: 10.1016/j.paid.2008.01.013

[ref18] Ekinci O. Albayrak Y. Ekinci A. (2011). Attention-deficit hyperactivity disorder, impulsivity, and suicidality: a dangerous triad. J. Affect. Disord. 134, 420–426.

[ref19] Elices M. Soler J. Feliu-Soler A. Carmona C. Tiana T. Pascual J. C.. (2017). Combining emotion regulation and mindfulness skills for preventing depression relapse: a randomized-controlled study. Borderline Person. Disord. Emot. Dysregul. 4, 1–9. doi: 10.1186/s40479-017-0064-6, PMID: 28690851 PMC5497384

[ref20] Fields S. Ramos A. Reynolds B. (2021). The multidimensional nature of impulsivity and its implications for psychopathology. Personal. Individ. Differ. 171:110434.

[ref21] Frank E. Prien R. F. Jarrett R. B. Keller M. B. Kupfer D. J. Lavori P. W.. (1991). Conceptualization and rationale for consensus definitions of terms in major depressive disorder: remission, recovery, relapse, and recurrence. Arch. Gen. Psychiatry 48, 851–855. doi: 10.1001/archpsyc.1991.01810330075011, PMID: 1929776

[ref22] Gonçalves S. F. Chaplin T. M. Turpyn C. C. Niehaus C. E. Curby T. W. Sinha R.. (2019). Difficulties in emotion regulation predict depressive symptom trajectory from early to middle adolescence. Child Psychiatry Hum. Dev. 50, 618–630. doi: 10.1007/s10578-019-00867-8, PMID: 30689145 PMC6589375

[ref9005] Gratz K. L. Roemer L. (2004). Multidimensional assessment of emotion regulation and dysregulation: Development, factor structure, and initial validation of the difficulties in emotion regulation scale. J. Psychopathol. Behav. Assess. 26, 41–54.

[ref9006] Gross J. J. (2013) Handbook of emotion regulation. Guilford publications.

[ref9007] Harvey P. D. Greenberg B. R. Serper M. R. (1989). The affective lability scales: development, reliability, and validity. J. Clin. Psychol. 45, 786–793. 2808736 10.1002/1097-4679(198909)45:5<786::aid-jclp2270450515>3.0.co;2-p

[ref23] Hershenberg R. Groshek K. Curley A. Goldstein T. R. (2020). Impulsivity in mood disorders: implications for relapse and recurrence. Clin. Psychol. Sci. Pract. 27:e12344.

[ref24] Høegh M. C. Melle I. Aminoff S. R. Olsen S. H. Lunding S. H. Ueland T.. (2022). Affective lability and social functioning in severe mental disorders. Eur. Arch. Psychiatry Clin. Neurosci. 272, 873–885. doi: 10.1007/s00406-022-01380-1, PMID: 35084540 PMC9279216

[ref25] Hopfinger L. Berking M. Bockting C. L. H. Ebert D. D. (2016). Emotion regulation mediates the effect of childhood trauma on depression. J. Affect. Disord. 198, 189–197. doi: 10.1016/j.jad.2016.03.050, PMID: 27018937

[ref26] Huh H. J. Kim K. H. Lee H.-K. Chae J. H. (2017). The relationship between childhood trauma and the severity of adulthood depression and anxiety symptoms in a clinical sample: the mediating role of cognitive emotion regulation strategies. J. Affect. Disord. 213, 44–50. doi: 10.1016/j.jad.2017.02.009, PMID: 28189964

[ref9008] International Telecommunication Union [ITU]. (2023). Youth Internet use: Facts and figures. (2023). Available at: https://www.itu.int/itu-d/reports/statistics/2023/10/10/ff23-youth-internet-use/

[ref9009] Kendler K. S. Prescott C. A. Myers J. Neale M. C. (2003). The structure of genetic and environmental risk factors for common psychiatric and substance use disorders in men and women. Arch. Gen. Psychiatry. 60, 929–937. doi: 10.1001/archpsyc.60.9.929 12963675

[ref9010] Kessler R. C. Petukhova M. Sampson N. A. Zaslavsky A. M. Wittchen H.-U. (2015). Twelve-month and lifetime prevalence and lifetime morbid risk of anxiety and mood disorders in the United States. Int. J. Methods Psychiatr. Res. 21, 169–184. doi: 10.1002/mpr.1359 PMC4005415 22865617

[ref27] Kroenke K. Spitzer R. L. Williams J. B. (2001). The PHQ-9: validity of a brief depression severity measure. J. Gen. Intern. Med. 16, 606–613. doi: 10.1046/j.1525-1497.2001.016009606.x, PMID: 11556941 PMC1495268

[ref9011] Lahey B. B. (2009). Public health significance of neuroticism. Am. Psychol. 64, 241–256. doi: 10.1037/a0015309 19449983 PMC2792076

[ref28] LeMoult J. Gotlib I. H. (2019). Depression: a cognitive perspective. Clin. Psychol. Rev. 69, 51–66. doi: 10.1016/j.cpr.2018.06.008, PMID: 29961601 PMC11884012

[ref30] Liu D. Y. Thompson R. J. (2017). Selection and implementation of emotion regulation strategies in major depressive disorder: an integrative review. Clin. Psychol. Rev. 57, 183–194. doi: 10.1016/j.cpr.2017.07.004, PMID: 28739277

[ref29] Liu Q. He H. Yang J. Feng X. Zhao F. Lyu J. (2020). Changes in the global burden of depression from 1990 to 2017: findings from the global burden of disease study. J. Psychiatr. Res. 126, 134–140. doi: 10.1016/j.jpsychires.2019.08.002, PMID: 31439359

[ref31] Marwaha S. Price C. Scott J. Weich S. Cairns A. Dale J.. (2018). Affective instability in those with and without mental disorders: a case control study. J. Affect. Disord. 241, 492–498. doi: 10.1016/j.jad.2018.08.046, PMID: 30149337

[ref32] Mennin D. S. Holaway R. M. Fresco D. M. Moore M. T. Heimberg R. G. (2007). Delineating components of emotion and its dysregulation in anxiety and mood psychopathology. Behav. Ther. 38, 284–302. doi: 10.1016/j.beth.2006.09.001, PMID: 17697853

[ref33] Miller J. D. Pilkonis P. A. (2006). Neuroticism and affective instability: the same or different? Am. J. Psychiatry 163, 839–845. doi: 10.1176/ajp.2006.163.5.839 16648325

[ref34] Navrady L. B. Ritchie S. J. Chan S. W. (2017). Intelligence and neuroticism in relation to depression and psychological distress: evidence from two large population cohorts. Eur. Psychiatry 43, 58–65. doi: 10.1016/j.eurpsy.2016.12.012, PMID: 28365468 PMC5486156

[ref35] Ngo T. T. Ruparel K. Loughead J. Gur R. C. (2011). Brain-behaviour relationships in impulsivity and depression. NeuroImage 54, S243–S249.

[ref36] Oliver M. N. Simons J. S. (2004). The affective lability scales: development of a short-form measure. Personal. Individ. Differ. 37, 1279–1288. doi: 10.1016/j.paid.2003.12.013

[ref37] Olmos A. Govindasamy P. (2019). A practical guide for using propensity score weighting in R. Pract. Assess. Res. Eval. 20:13.

[ref9012] Ormel J. Jeronimus B. F. Kotov R. Riese H. Bos E. H. Hankin B. (2013). Neuroticism and common mental disorders: Meaning and utility of a complex relationship. Clin. Psychol. Rev. 33, 686–697. 23702592 10.1016/j.cpr.2013.04.003 PMC4382368

[ref38] Patton J. H. Stanford M. S. Barratt E. S. (1995). Factor structure of the Barratt impulsiveness scale. J. Clin. Psychol. 51, 768–774. doi: 10.1002/1097-4679(199511)51:6<768::AID-JCLP2270510607>3.0.CO;2-1 8778124

[ref9013] Rudolph K. D. Flynn M. Abaied J. L. (2014). “A developmental perspective on interpersonal theories of youth depression,” in Handbook of depression in adolescents. eds. Nolen-Hoeksema S. Hilt L. M. (New York: Routledge) 79–102.

[ref39] Saddichha S. Schuetz C. (2014). Impulsivity in remitted depression: a meta-analytical review. Asian J. Psychiatr. 9, 13–16. doi: 10.1016/j.ajp.2014.02.003 24813029

[ref40] Sperry S. H. Walsh M. A. Kwapil T. R. (2020). Emotion dynamics concurrently and prospectively predict mood psychopathology. J. Affect. Disord. 261, 67–75. doi: 10.1016/j.jad.2019.09.076 31600589

[ref41] Spitzer R. L. Kroenke K. Williams J. B.. (2006). A brief measure for assessing generalized anxiety disorder: the GAD-7. Arch. Intern. Med. 166, 1092–1097. doi: 10.1001/archinte.166.10.1092, PMID: 16717171

[ref42] Teicher M. H. Samson J. A. Anderson C. M. Ohashi K. (2016). The effects of childhood maltreatment on brain structure, function and connectivity. Nat. Rev. Neurosci. 17, 652–666. doi: 10.1038/nrn.2016.111, PMID: 27640984

[ref43] Visted E. Vøllestad J. Nielsen M. B. Schanche E. (2018). Emotion regulation in current and remitted depression: a systematic review and meta-analysis. Front. Psychol. 9:756. doi: 10.3389/fpsyg.2018.00756, PMID: 29867700 PMC5968125

[ref44] Whiteside S. P. Lynam D. R. (2001). The five-factor model and impulsivity: using a structural model of personality to understand impulsivity. Personal. Individ. Differ. 30, 669–689. doi: 10.1016/S0191-8869(00)00064-7

[ref45] Zwicker A. Drobinin V. MacKenzie L. E.. (2020). Affective lability in offspring of parents with major depressive disorder, bipolar disorder and schizophrenia. Eur. Child Adolesc. Psychiatry 29, 445–451. doi: 10.1007/s00787-019-01355-z, PMID: 31172297

