# Supplementary material for: The impact of personality traits on the return of major depression: a case–control study
Source: Front Psychol. 2025 Mar 31;16:1454673. doi: 10.3389/fpsyg.2025.1454673 (PMC11994710; doi:10.3389/fpsyg.2025.1454673)
Supplement: Supplementary file 1 [file Data_Sheet_1.docx]

***Supplementary Material***

**The impact of personality traits on the return of major depression: a case-control study**

Nada Altaweel^*^, Rachel Upthegrove, Steven Marwaha

*** Corresponding author:** Nada Altaweel

Email: naaltaweel@pnu.edu.sa


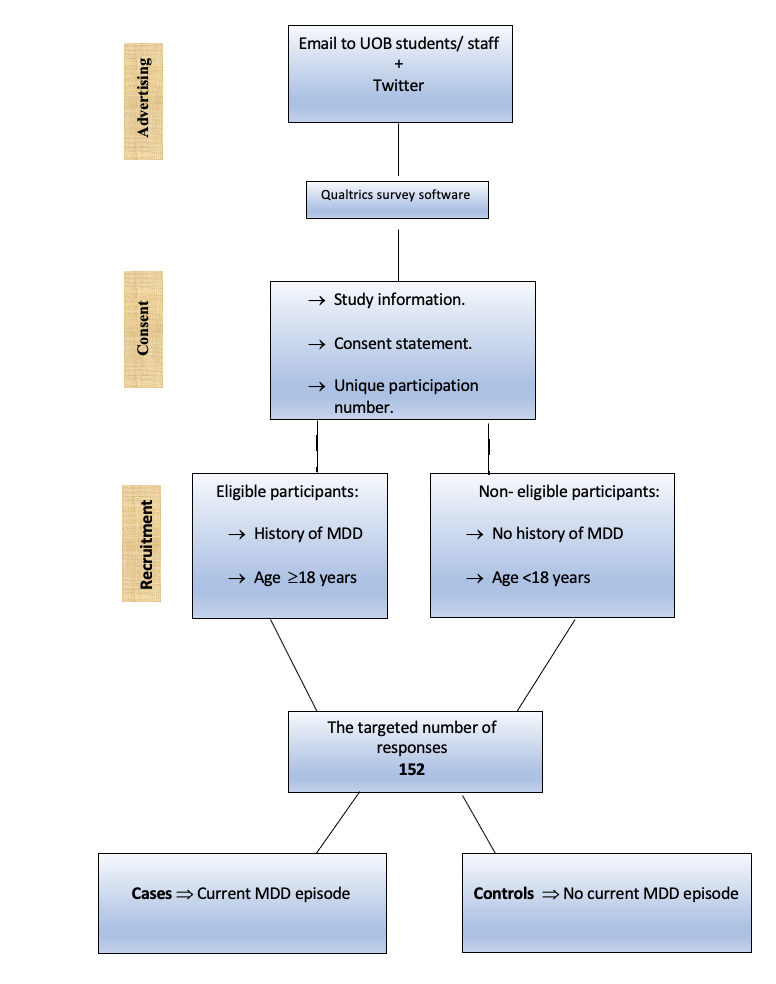


diagram 1: Recruitment procedure


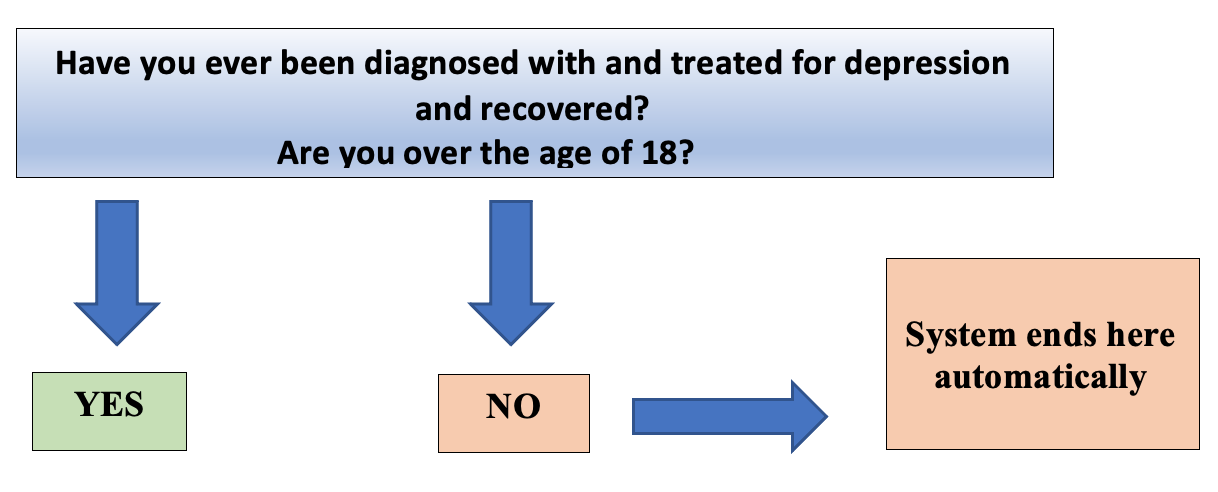


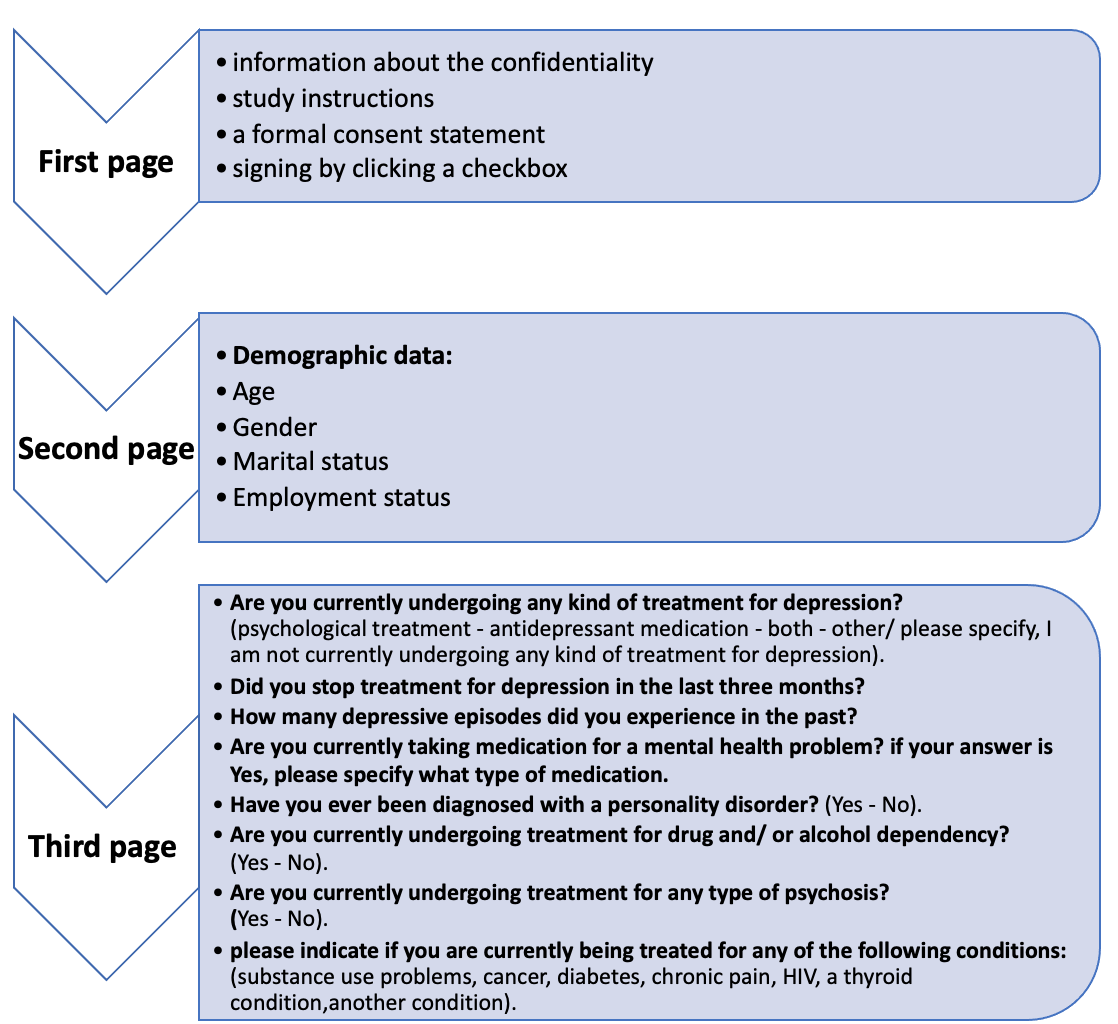


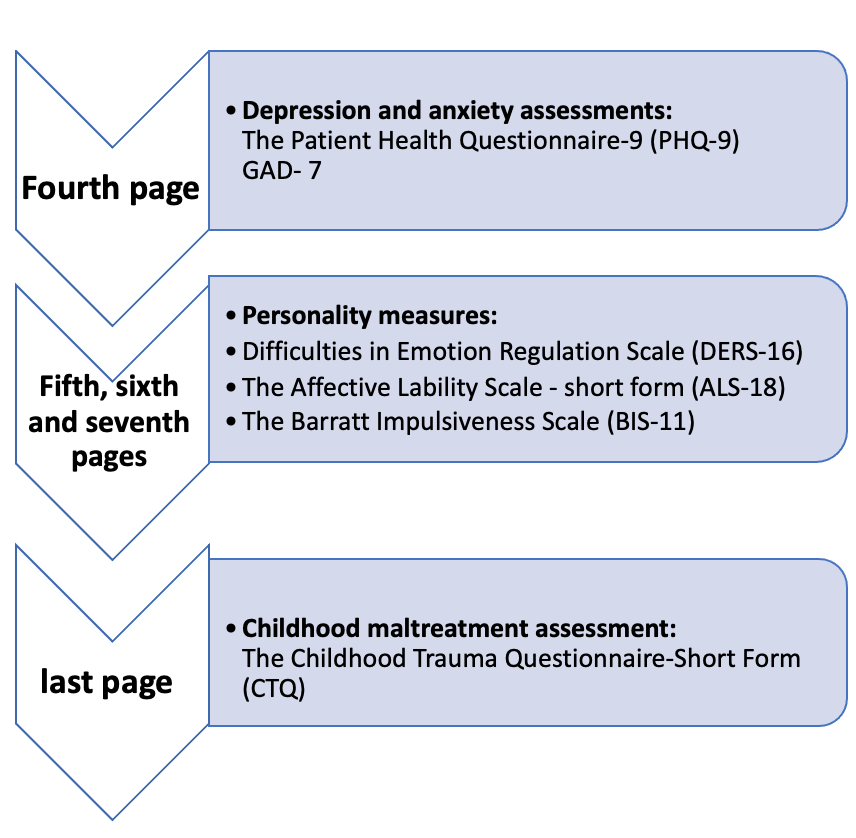


diagram 2: order of the study questionnaires
